# Qubit crossover in the endohedral fullerene Sc_3_C_2_@C_80_†
†Electronic supplementary information (ESI) available. See DOI: 10.1039/c7sc03749j


**DOI:** 10.1039/c7sc03749j

**Published:** 2017-11-02

**Authors:** Zheng Liu, Bo-Wei Dong, Hai-Bing Meng, Mei-Xing Xu, Tai-Shan Wang, Bing-Wu Wang, Chun-Ru Wang, Shang-Da Jiang, Song Gao

**Affiliations:** a National Laboratory for Molecular Sciences , State Key Laboratory of Rare Earth Materials Chemistry and Applications , College of Chemistry and Molecular Engineering , Peking University , Beijing 100871 , P. R. China . Email: jiangsd@pku.edu.cn ; Email: gaosong@pku.edu.cn; b Key Laboratory of Molecular Nanostructure and Nanotechnology , Beijing National Laboratory for Molecular Sciences , Institute of Chemistry , Chinese Academy of Sciences , Beijing 100190 , P. R. China . Email: crwang@iccas.ac.cn

## Abstract

The core–shell structure of endohedral fullerenes results in good protection of the encapsulated spin carriers from the environment. In this research, the quantum coherence behavior of the endohedral fullerene Sc_3_C_2_@C_80_ in CS_2_ solution is characterized from 5 K to room temperature. Below the critical temperature of around 140 K, the inner group is hindered, and the EPR spectrum consists of a single broad line. The spin carriers display a maximum phase memory time of 17.2(7) μs at 10 K. In the high temperature region, the inner group is mobile, and the EPR spectrum consists of 22 homogeneously broadened lines due to isotropic hyperfine coupling. The maximum phase memory time for each transition is around 139(1) ns at 200 K which allows arbitrary superposition state manipulations to be performed. This research demonstrates that Sc_3_C_2_@C_80_ displays temperature-crossover behaviour due to weak interaction between the Sc_3_C_2_ core and the C_80_ shell.

## Abstract 2

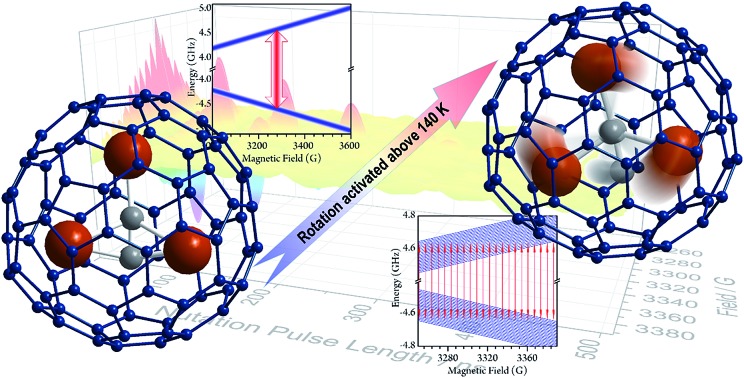
The qubit crossover behavior of the endohedral fullerene Sc_3_C_2_@C_80_ in CS_2_ solution is characterized from 5 K to room temperature.

## Introduction

Quantum computation innovates the conventional way of computing.^1^ The basic units for quantum computers are quantum bits (qubits). For the purpose of computation application, long phase memory times are required for quantum manipulation. Besides the various qubits involved in previous research, chemists have demonstrated that magnetic molecules can also be used as potential qubits. Several groups have successfully built qubits using paramagnetic molecules, such as Cr_7_Ni,^2^ single-molecule magnets,^3^,^4^ mononuclear transition ion complexes^5^–^18^ and endohedral fullerenes.^19^

Endohedral fullerenes are core–shell structure molecules synthesised by encapsulating atoms in fullerene cages. This is an efficient way to isolate the core from the environment so as to stabilize the inner chemical stability and even enhance their physical properties. In previous research, the electron spin suffers rapid decoherence due to environmental effects. It is straightforward to protect the qubit in the fullerene, resulting in enhancement of the quantum coherence behavior. The carbon cage offers many advantages in comparison to other systems. The fullerene shell can work as a closed form of protection from the environment.^20^ Moreover, the encapsulation structure is maneuverable and visible for single molecule device manipulation.^21^ As an example, N@C_60_ is the first well-investigated endohedral fullerene qubit with a distinctive electronic structure and quantum coherence behaviour. There have been many reports on the synthesis of endohedral fullerenes.^22^–^24^

A further interesting feature of endohedral fullerenes is that the interaction between the core and shell is weak and more isotropic, indicating that motion of the inner group is possible. This is very different from traditional coordination compounds, which possess rigid chemical bonds and are normally inflexible with respect to temperature variation. It is reasonable to predict that the temperature dependence of the structure is able to induce transformation of the physical properties. A good example is spin crossover behavior which is a spin state transformation resulting from structure alternation induced by temperature and/or light.^25^ In previous research of molecular qubits, chemists have mainly focused on coordination compounds, whose rigid chemical bonds cause uniform quantum coherence behavior in the investigated temperature range.

Lots of carbide cluster metallofullerenes and their structures and properties have been well investigated before.^26^ In this study we encapsulated a Sc_3_C_2_ cluster in a C_80_ fullerene. Our research results demonstrate that the electron spin, in the presence of three nuclear spin carriers (^45^Sc, *I* = 7/2), is well protected in the carbon cage, resulting in a quantum phase memory time of 17.2(7) μs at 10 K which can be enhanced to 68.0(4) μs. More interestingly, this molecular qubit displays an abrupt change in quantum coherence properties upon temperature variation that arises from the core motion.

## Results and Discussion

**Fig. 1:**
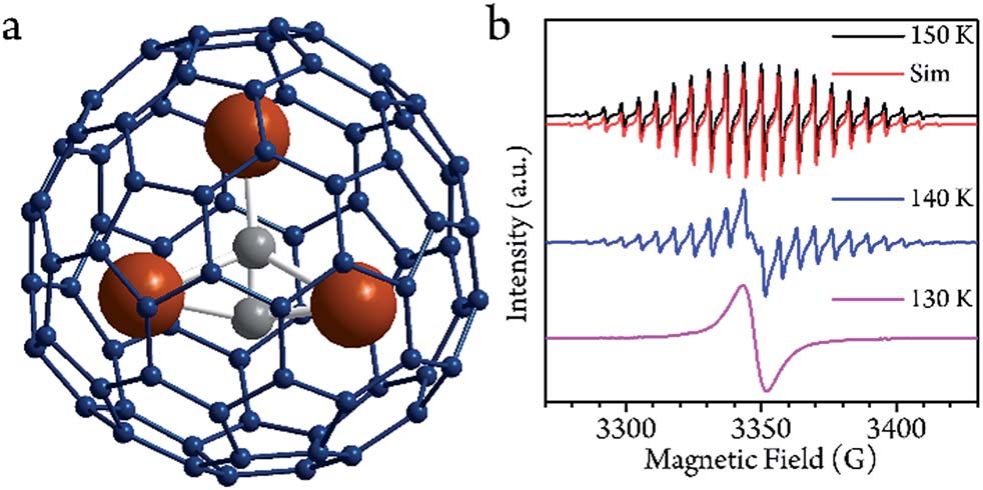
(a) The optimized structure of the Sc_3_C_2_@C_80_ based on DFT calculations. (b) cw-EPR spectra from 130 to 150 K in CS_2_ solution. The spectrum at 150 K can be reproduced with the following parameters: *g*_iso_ = 2.00 and *A*_iso_ = 18.10 MHz. The simulation was performed by the EasySpin toolbox in the Matlab software.

Sc_3_C_2_@C_80_ was originally considered as Sc_3_@C_82_,^27^,^28^ but further NMR investigation indicated that there are two types of carbon atoms in one molecule.^29^ The structure was first determined by single crystal X-ray diffraction analysis of the derivative molecule.^29^ Further investigation has provided more evidence of the structure.^30^ Three ^45^Sc ions surround the C_2_^3–^ group, and are encapsulated by a C_80_ cage (Fig. 1a). The two types of conformational isomer with the C_2_ lying inside and outside of the Sc_3_ plane have a slight energy difference.^29^,^31^ DFT calculations demonstrated that the charge distribution of the molecule can be expressed by the formula (Sc^3+^)_3_(C_2_)^3–^@(C_80_)^6–^, and the electron spin 1/2 delocalizes in the Sc_3_C_2_ cluster.

### cw-EPR characterization

The cw-EPR spectra of Sc_3_C_2_@C_80_, which were solved in a low concentration of CS_2_ (0.02 mmol L^–1^, see ESI†) from 150 K to 130 K, are plotted in Fig. 1b. To ensure the temperature stability, all of the measurements were performed after a 30 min delay once the target temperatures were achieved. At 150 K, which is below the melting point of CS_2_ (161 K), a 22-fold feature with a linewidth of 1.4 G could be resolved. This spectrum could be reproduced by the effective spin Hamiltonian*Ĥ* = *gμ*_B_*Bŝ* + *AŝÎ*,where the first term represents the Zeeman effect of the electron spin, and the second one represents the hyperfine coupling between the electron spin and the three ^45^Sc nuclei. The best simulation was found with isotropic parameters of *g* = 2.00 and *A* = 18.10 MHz, which are identical to previous studies.^28^,^32^,^33^ Upon cooling, 22 lines gradually disappeared and were replaced with a single 12 G wide line below 130 K.^28^ This abrupt transformation of the cw-EPR spectra was largely due to the Sc_3_C_2_ core motion. At above 140 K, mobility of the core was possible due to the weak interaction between the core and shell. According to previous works, the energy barrier of the rotation is less than 5 kcal mol^–1^,^34^ and the first principles molecular dynamics of the Sc_3_C_2_@C_80_ simulation gives the rotation speed of 3.5 ps at 150 K.^35^ The free mobility of the core affords three equivalent Sc^3+^ ions and the hyperfine coupling is therefore isotropic. This motion was independent from the solution freezing according to the EPR spectrum, in which no obvious line-width changing is observed crossing the melting point at 161 K. At below 140 K, the core motion was restricted due to the suppressed thermal fluctuation. This structure alternation led to the hyperfine coupling tensor variation and was no longer isotropic, resulting in the observed broad single line.

### Qubit behavior in the low temperature region

Disparate quantum coherence behaviors in the high and low temperature regions could be observed, which were also due to the core motion effect. In the low temperature region, the spin-lattice relaxation time (*T*_1_) and phase-memory time (*T*_M_) were measured by inversion recovery (π–*T*–π/2–*τ*–π–*τ*–echo) and Hahn echo decay, respectively. The *T*_1_ decreased upon warming across the whole temperature range, indicating that the thermal process dominates the longitudinal relaxation. On the contrary, the *T*_M_ remained nearly constant below 40 K (13.6(4) μs at 5 K, a maximum of 17.2(7) μs at 10 K and 10.6(5) μs at 20 K), and started to decrease upon warming.

The decoherence of the electron spin superposition state can be caused by nuclear spin, phonon or intermolecular electron dipolar interaction.^36^,^37^ According to the concentration dependence of the *T*_M_, the molarity (0.02 mmol L^–1^) was low enough in comparison to that of previous publications.^2^,^7^ The intermolecular dipolar–dipolar interaction is therefore regarded as a minor effect in the decoherence behavior. As to the effects of the phonon, we can consider it in two regions. At above 40 K, the *T*_M_ dropped upon warming with a decrease of the *T*_1_. The *T*_M_ was found to be around 1/6 of the *T*_1_. This is a strong indication that, in the temperature region above 40 K, the spin-phonon interaction was the main effect that caused decoherence of the quantum superposition. However, the *T*_m_ remained nearly constant with respect to the variation of the *T*_1_ at below 40 K. From a quantitative point of view, 1/*T*_M_ is in the order of 10^5^ s^–1^ which is vastly larger than that of 1/*T*_1_ (about 10^2^ s^–1^). Therefore, the phonon effect contributes very little to the decoherence herein.^38^ The evidence leads to the conclusion that the decoherence of the electron spin at below 40 K was largely caused by surrounding nuclear spin carriers.

The nuclear spins around the electron spin act as a local magnetic field (Overhauser field, *B*_hf_). The real magnetic field felt by the electron is the combination of the magnetic field of the spectrometer (*B*_0_) and the fluctuating Overhauser field. This effect leads to inhomogeneous broadening, and reduces the *T*_M_. Herein, we demonstrate that the fluctuating Overhauser effect is suppressed by the dynamic decoupling in the present molecule, where a train of refocusing pulses are applied before the echo detection.^39^ The following CPMG-*n* pulse sequence (π/2–*τ*–(π – 2*τ*)_*n*–1_–π–*τ*–echo) was applied to the system, where *n* denotes the number of the inversion π pulses. As previously discussed, the fluctuating Overhauser field is the major effect that causes the decoherence. Therefore, dynamic decoupling enhanced the *T*_M_ effectively. When the CPMG-1 (Hahn pulses) was applied, the *T*_M_ was only 13 μs. When there were more refocusing pulses, they effectively increased the *T*_M_ to 67 μs with 32 π pulses. Nevertheless, we were unable to keep increasing *n* because the echo intensity also decreased largely at the same time. The applied pulses can never be perfect. Therefore, the error of the refocusing pulses would accumulate and finally destroy the quantum phase coherence. One solution is to employ more effective dynamic decoupling sequences.^40^–^42^

The electron spin echo envelope modulation (ESEEM) measurement was taken at 5 K (see ESI†). However, the anisotropic hyperfine interaction involving three different Sc^3+^ ions would result in too many parameters to determine. Moreover, the Hilbert space is of 1024-dimension, making the simulation difficult.

### Qubit behavior in the high temperature region

**Fig. 2:**
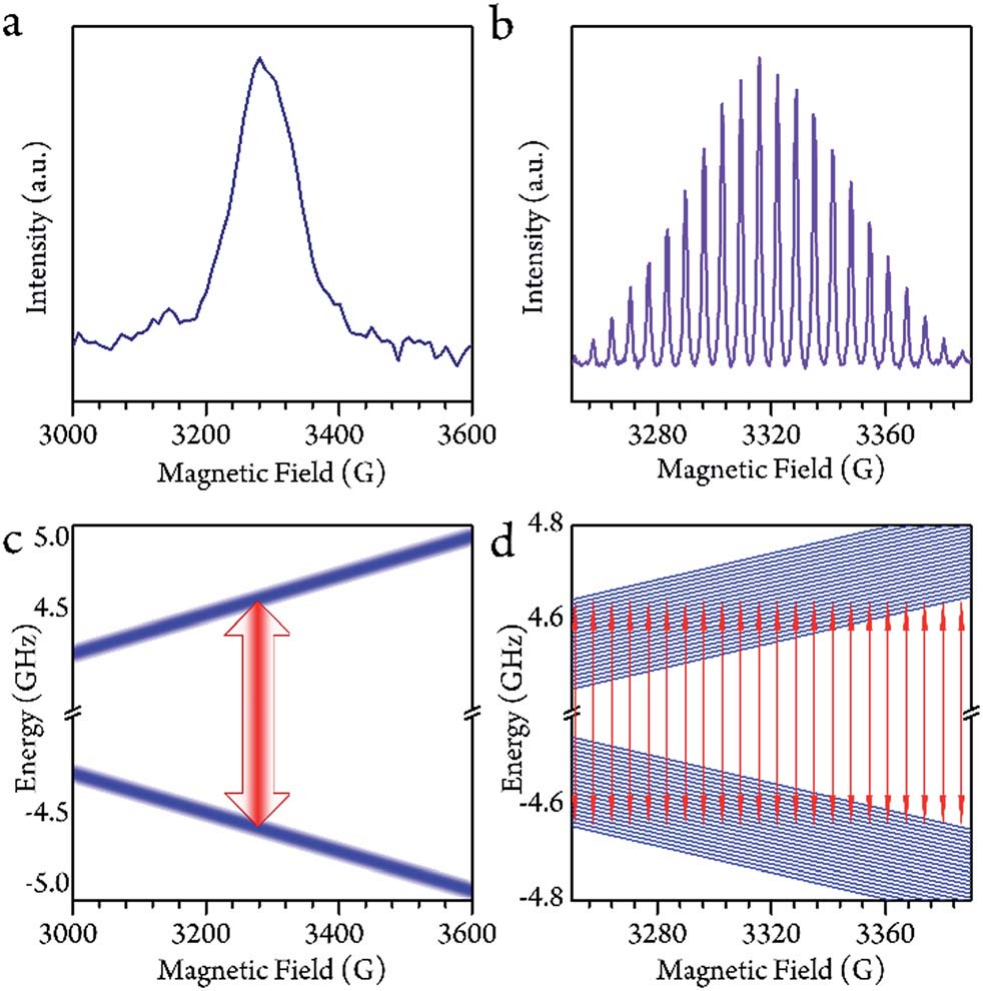
(a and b) The echo detected field swept (EDFS) spectra of the sample at 5 K and 200 K, respectively. Their corresponding energy levels and transitions are indicated in parts c and d. In the low temperature region (a and c) the energy diagram is represented as a pseudo-two-level picture; in the high temperature region (b and d), the 22-transition lines are well resolved.

**Fig. 3:**
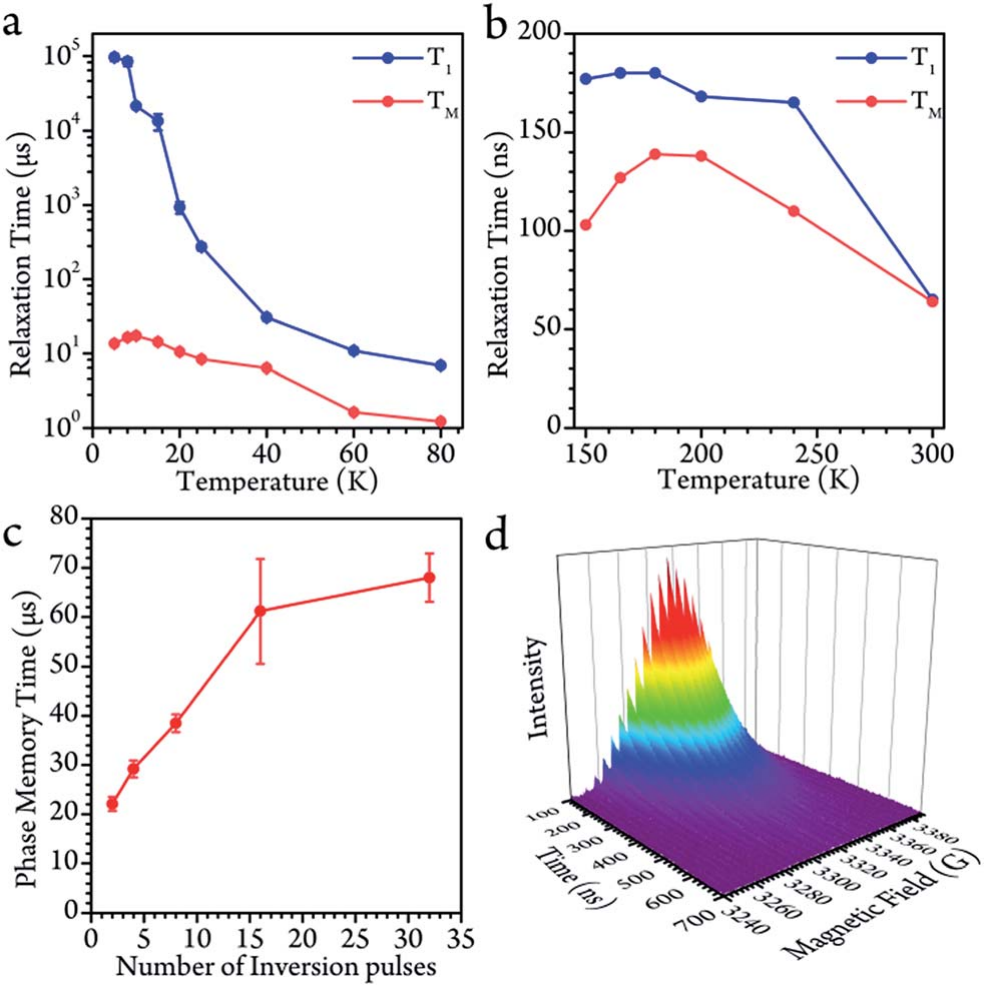
(a and b) The longitudinal relaxation time (*T*_1_) and phase memory time (*T*_M_) in the low and high temperature regions. The spin echo signal is hard to record from 80 to 150 K. (c) The phase memory time enhanced by dynamic decoupling with various numbers of inversion pulses. (d) The spin echo decays as a function of the magnetic field.

As the system warmed up from the base temperature, the spin echo intensity decreased. At above 80 K, the intensity of the spin echo was largely reduced, was not resolvable in environmental noise and became hardly visible. Nevertheless, the spin echo signals suddenly appeared at 150 K. The echo detected field swept (EDFS) spectrum was measured at 200 K and clearly shows the 22-line feature, as shown in Fig. 2b. Every individual line in the cw-EPR spectrum is of Lorentzian shape, indicating that the *T*_M_ was not affected by nuclei spin. The major effect that limited the spin coherence time was the rapid longitudinal relaxation. The *T*_M_ and *T*_1_ could be recorded from 150 K to room temperature, as shown in Fig. 3b. In this temperature range, the *T*_1_ decreased as the temperature increased, while the *T*_M_ showed a maximum value of 139(1) ns at around 200 K. This was probably caused by the fact that the ^13^C located at the outer shell with a natural abundance (1.07%) decoherence effect was averaged when the solution melted above 161 K and the fullerene cages started to rotate rapidly. This analysis reveals that the 22 transitions concerning different nuclear spin momentum behave with a similar phase memory time at a certain temperature while higher nuclear spin moments will slightly accelerate the phase decoherence. This finding is consistent with previous research.^43^

### Rabi oscillation

To check for the possibility of putting the spin in an arbitrary superposition state, a nutation experiment was performed in both temperature regions. In the present hyperfine coupled system, the superposition state involved only the electron spin states |±1/2To check for the possibility of putting the spin in an arbitrary superposition state, a nutation experiment was performed in both temperature regions. In the present hyperfine coupled system, the superposition state involved only the electron spin states |±1/2〉, as the variation of the nuclear spin momentum is forbidden in the EPR transition. An oscillating magnetic field (, as the variation of the nuclear spin momentum is forbidden in the EPR transition. An oscillating magnetic field (*B*_1_) from the microwave was applied to drive the spin rotation in the plane normal to the *B*_1_ field. The tip angle (*θ*) depends on the applied strength and time of the *B*_1_ field.

**Fig. 4:**
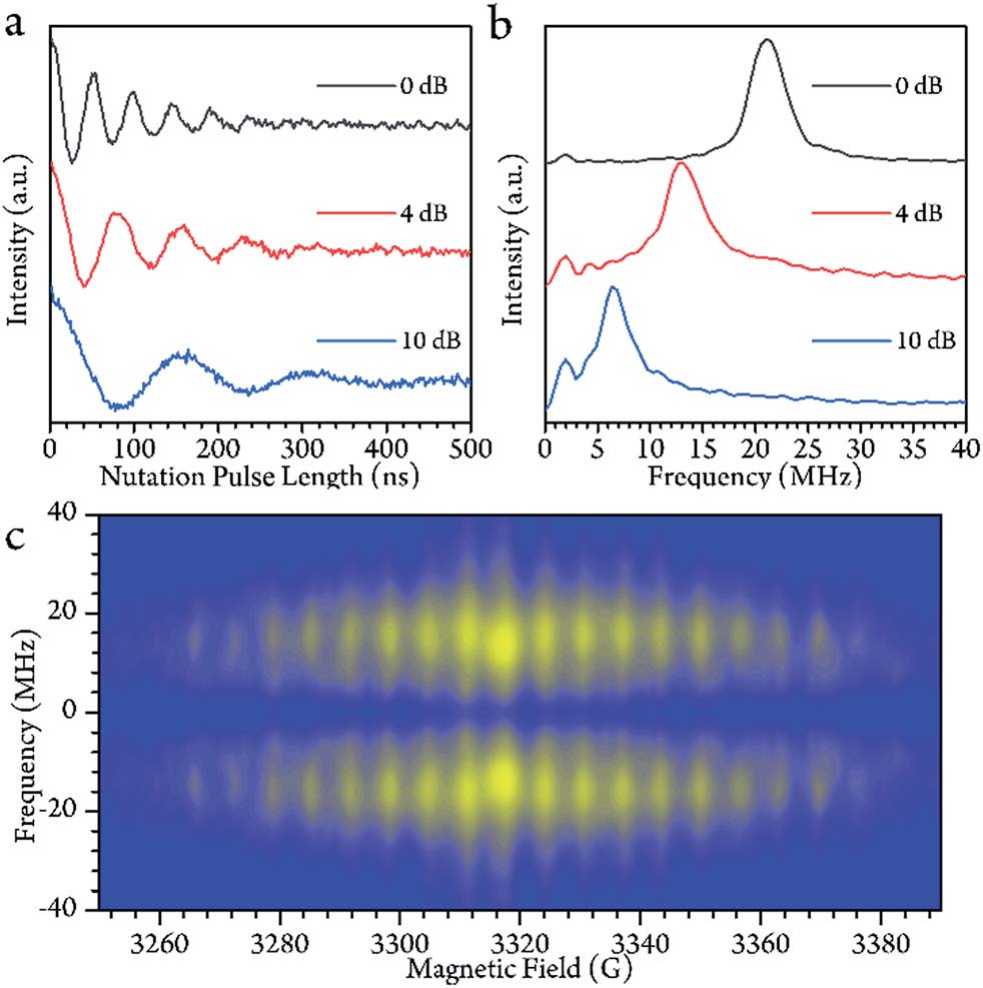
(a) The Rabi oscillation measured at the center of the field at 200 K with various powers of microwaves. The value of dB indicates the microwave attenuated from the full power (300 W) of the solid-state amplifier. (b) The Fourier transform of (a). The oscillation frequency behaves as a linear function of the microwave *B*_1_ field. (c) The Fourier-transformed Rabi oscillation as a function of the magnetic field *B*_0_ at 200 K. The Rabi frequencies remain constant over the whole field range.

In the high temperature region, we detected magnetization of the spin, and observed that it oscillated with a certain period as the length of the driven pulse, known as Rabi oscillation. The frequency (*Ω*_R_) is proportional to the driven magnetic field.^38^ The nutation experiment was performed at a variety of temperatures, and the Fourier transform result is shown in Fig. 4c and the ESI.† The oscillation decays mainly resulted from decoherence of the spin. Further investigation of the field scan also showed that nutation was available for all of the transitions (
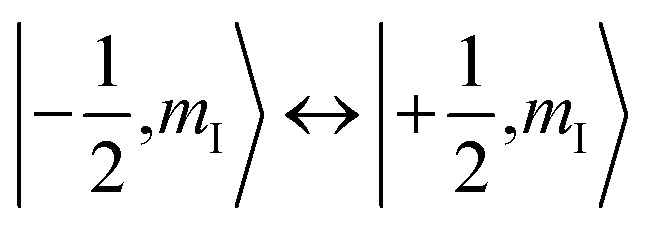
). The whole spectrum of Sc_3_C_2_@C_80_ is approximately 135 G, offering the possibility to excite any transitions with an arbitrary wave generator (AWG) of 400 MHz bandwidth when the external *B*_0_ field is set to the center of the spectrum. Therefore, the arbitrary superposition state of basis 
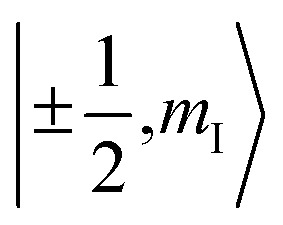
 with any specific *m*_I_ value can be manipulated without varying the *B*_0_ field.

In the low temperature region, however, the Rabi oscillation is hardly visible. This is probably due to the degrees of freedom with respect to the electron and nuclear being strongly linked to each other since the core motion is frozen at below 140 K. This situation changes when the core mobility is obvious.

## Conclusion

Different from previously reported molecular qubits, Sc_3_C_2_@C_80_ is a dual-behavioral qubit. Its quantum coherence behaviors are very different below and above the critical temperature of around 140 K. In the low temperature region, the longest quantum phase memory time is nearly 20 μs. However, only one broad line is resolved and Rabi oscillation is hardly observable. In contrast, all of the 22 transitions’ quantum coherence is evidently observed and the arbitrary superposition state of the |±1/2 is a dual-behavioral qubit. Its quantum coherence behaviors are very different below and above the critical temperature of around 140 K. In the low temperature region, the longest quantum phase memory time is nearly 20 μs. However, only one broad line is resolved and Rabi oscillation is hardly observable. In contrast, all of the 22 transitions’ quantum coherence is evidently observed and the arbitrary superposition state of the |±1/2〉 basis with any specific nuclear spin momentum is accessible in the high temperature region. The phase memory time at high temperature is two orders shorter than that of the low temperature region. Considering the weak polarization, this crossover cannot be explained by the thermal population variation of the energy levels, but is probably due to the mobility of the inner group. Since the rotational correlation time of the magnetic core Sc basis with any specific nuclear spin momentum is accessible in the high temperature region. The phase memory time at high temperature is two orders shorter than that of the low temperature region. Considering the weak polarization, this crossover cannot be explained by the thermal population variation of the energy levels, but is probably due to the mobility of the inner group. Since the rotational correlation time of the magnetic core Sc_3_C_2_@C_80_ (3.5 ps)^35^ is very short and the molecule possesses a large mass compared to that of dinuclear gas molecules, the spin-rotational interaction induced EPR line splitting is not observable.^44^ However, this environmental effect originating from the core rotation indeed causes decoherence of the quantum phase and dramatically increases upon warming.

For the purpose of quantum information processing, the quantum gates are of fundamental importance, in which the controlled-NOT gate requires coupled qubits, either between electron–electron or electron–nuclear spin. However, this type of coupling could lead to rapid decoherence. By encapsulating the electron and nuclear spin carriers in the fullerene, the qubit is very well protected by the carbon cage. In the presence of three surrounding large nuclear spins, the quantum coherence can still survive for tens of microseconds, proving that the strategy is effective. Besides, the 22 transitions can be well resolved in the high temperature region. The short phase memory time is mainly limited by longitudinal relaxation. Strategies to enhance the *T*_1_ are then demanded.

Taking advantage of the carbon cage, endohedral fullerenes can be placed on the surface of or inside the carbon nanotube to assemble in dimensions. It is therefore reasonable to believe that Sc_3_C_2_@C_80_ could act as elements of quantum information processing, building up to quantum gates by employing the electron spin and the relevant scandium nuclei spins. For further investigation, pulse ENDOR can be used to generate an entangled state and quantum gate. An individual molecule investigation on the surface to build up quantum devices is also of general interest.

## Methods

### Synthesis

Sc_3_C_2_@C_80_ was synthesized and purified as in literature methods.^27^

### EPR measurements

The Sc_3_C_2_@C_80_ was dissolved in a CS_2_ solution for EPR experiments with a concentration of 0.02 mmol L^–1^ determined by the Bruker spin counting method (see ESI†). cw-EPR spectra were measured on a Bruker Elexsys E580 spectrometer with a super-high sensitivity probe head (*ω* = 9.37 GHz). Pulsed EPR data were collected on the same system by an MS-3 cavity (*ω* = 9.28 GHz). The cw-EPR spectra were simulated by the EasySpin toolbox based on Matlab. The low-temperature environment was achieved by liquid helium cryostats (ESR900 for cw and CF935 for pulse) produced by Oxford Instruments. The measurement temperatures were stabilized for 30 min before the measurements were carried out. The signal of the pulsed-EPR experiments was collected by integrating the Hahn echo (π/2–*τ*–π–*τ*–echo) with *τ* = 100 and 150 ns at a high and low temperature range, respectively. The *T*_1_ values were measured by the inversion recovery method (π–*T*–π/2–*τ*–π–*τ*–echo) with 16-step phase cycling. The *T*_m_ values were obtained by increasing the *τ* value of the Hahn echo sequence with 16-step phase cycling. Dynamic decoupling measurements were carried out by the CPMG sequence (π_*x*_/2–*τ*–(π_*y*_ – 2*τ*)_*n*_–π_*y*_–*τ*–echo) with 16-step phase cycling. The π/2 and π pulse lengths in EDFS, *T*_1_ and *T*_m_ measurements, were 8 and 16 ns, respectively. Nutation experiments were carried out with a standard sequence (*t*_p_–*T*–π/2–*τ*–π–*τ*–echo), where *T* > 5*T*_m_. The π/2 pulse lengths were adjusted to 10, 16 and 32 ns by 0 dB, 4 dB and 10 dB attenuation.

## Author contributions

Z. L. and B.-W. D. performed the EPR measurements, assisted by M.-X. X. Z. L. analyzed the EPR data. H.-B. M. synthesized the sample in support of T.-S. W. and C.-R. W. The project was conceived by S. G. and S.-D. J. S.-D. J. designed the experiments and wrote the manuscript. All of the authors revised the manuscript.

## Conflicts of interest

The authors declare no conflict of interest.

## Supplementary Material

Supplementary information
