# Supplementary material for: Qubit crossover in the endohedral fullerene Sc3C2@C80
Source: Chem Sci. 2017 Nov 2;9(2):457–62. doi: 10.1039/c7sc03749j (PMC6113862; doi:10.1039/c7sc03749j)
Supplement: Supplementary file 1 [file SC-009-C7SC03749J-s001.pdf]

Electronic Supplementary Material (ESI) for Chemical Science.
This journal is © The Royal Society of Chemistry 2017

Supporting Information

Qubit Crossover in the Endohedral Fullerene $Sc_3C_2@C_{80}$

Zheng Liu[1], Bo-Wei Dong[1], Hai-Bing Meng[2], Mei-Xing Xu[1], Tai-Shan Wang[2], Bing-Wu Wang[1], Chun-Ru Wang[2*], Shang-Da Jiang[1*] and Song Gao[1*]

[1] National Laboratory for Molecular Sciences, State Key Laboratory of Rare Earth Materials Chemistry and Applications, College of Chemistry and Molecular Engineering, Peking University, Beijing 100871, P. R. China

[2] Key Laboratory of Molecular Nanostructure and Nanotechnology, Beijing National Laboratory for Molecular Sciences, Institute of Chemistry, Chinese Academy of Sciences, Beijing 100190, P. R. China

List of Supporting Information

SI1 Sample preparation and physical measurement information
SI2 Continuous wave EPR measurements
SI3 Relaxation measurements at high temperature region
SI4 Relaxation measurements at low temperature region
SI5 ESEEM measurements at low temperature region

SI1 Sample preparation and physical measurement information

$Sc_3C_2@C_{80}$ sample was solved in CS_2 (purchased from J&K Scientific, Super Dry Solvents) in a 3-mm quartz EPR tube, then froze and pumped for 5 times to exclude oxygen. The concentration of $Sc_3C_2@C_{80}$ is determined by Bruker® spin counting by calculating using the height and diameter of the sample and the cavity quality factor Q at room temperature.(Eaton, G. R.; Eaton, S. S.; Barr, D. P.; Weber, R. T., *Quantitative EPR*. Springer Vienna: 2010.)

Continuous wave (cw) EPR measurements were performed on a Bruker E580 spectrometer using ER 4122 SHQE high sensitive EPR cavity. Oxford ESR900 cryostat was used for temperature control.

Pulse EPR measurement were performed on a Bruker E580 spectrometer using ER 4118 X-MS3 cavity. Oxford CF935 cryostat was used for temperature control.

SI2 Continuous wave EPR measurements

Continuous wave EPR measurements were performed at various temperatures from 130 K to 150 K. The microwave frequency is at 9.367902 GHz. The spectra are shown in **Figure S2 (black)**. The best simulation for experiment data at 150 K are shown in **Figure S2 (red)**. The best parameters for simulation is g_{iso} = 2.00 , A_{iso} = 18.10 MHz at 150 K.

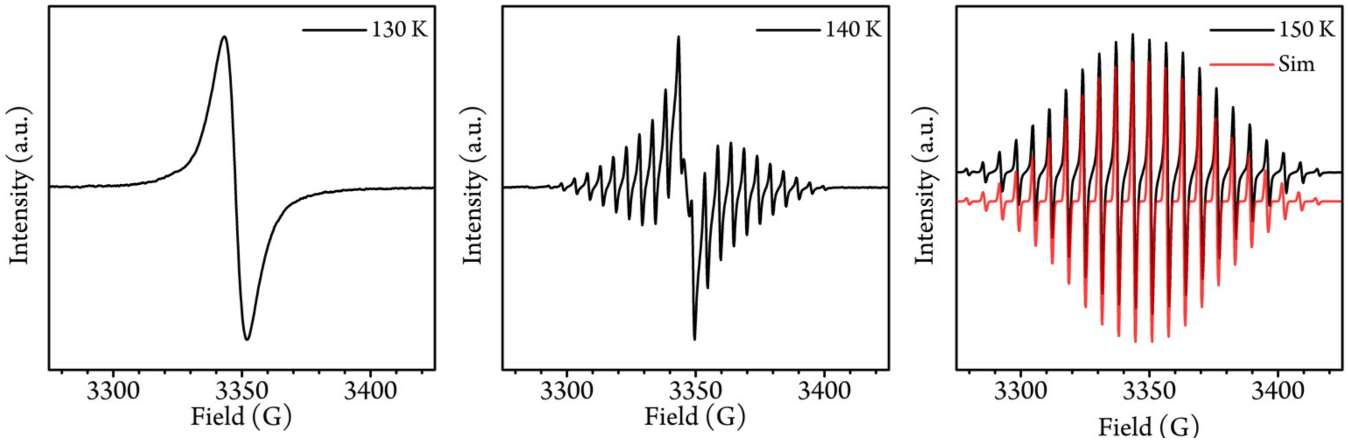

Figure S2. cw-EPR measurements on Bruker E580 at different temperature in CS_2 solution

SI3 Relaxation measurements at high temperature region

1. Spin-lattice relaxation (T_1) at different temperatures

   T_1 is measured using inverse-recovery method. The pulse sequence is π-T-π/2-τ-π-τ-echo with fixed τ and varied T. We use π/2 = 8 ns and π = 16 ns pulse for the measurement. τ = 100 ns is chosen for detection. 16-step phase cycling was used to eliminate the contribution of unwanted free-induction decay (FID) and echo signals. The experiment data are shown in **Table S3-1.**

Table S3-1. T_1 experiment data measured at different temperatures

Tempreature	T_1 value	Tempreature	T_1 value	Tempreature	T_1 value
150 K	177(1) ns	165 K	180(1) ns	180 K	180(1) ns
200 K	168(1) ns	240 K	165(1) ns	298 K	66(1) ns

2. Phase memory time (T_M) at different temperature

   T_M is measured using Hahn-echo decay method. The pulse sequence is π/2-τ-π-τ-echo with τ varies. We use π/2 = 8 ns and π = 16 ns pulse for the measurement. τ = 100 ns is chosen for initial interval. 16-step phase cycling was used to eliminate the contribution of unwanted free-induction decay (FID) and echo signals. The experiment data are shown in **Table S3-2.**

Table S3-2. T_M experiment data measured at different temperatures

Tempreature	T_M value	Tempreature	T_M value	Tempreature	T_M value
150 K	98(1) ns	165 K	128(1) ns	180 K	139(1) ns
200 K	139(1) ns	240 K	111(1) ns	298 K	75(2) ns

3. Phase memory time (T_M) at different magnetic field

   T_M is measured using Hahn-echo decay method. The pulse sequence is π/2-τ-π-τ-echo with τ varies. We use π/2 = 8 ns and π = 16 ns pulse for the measurement. τ = 100 ns is chosen for initial interval. 16-step phase cycling was used to eliminate the contribution of unwanted free-induction decay (FID) and echo signals. The experiment data are shown in **Table S3-3.** The first and the last transitions are hardly observable due to the weak signal.

Table S3-3. T_M experiment data measured at magnetic field

Magnetic Field	TM value	Magnetic Field	TM value	Magnetic Field	TM value
3257.6 G	119(1) ns	3264.1 G	126(1) ns	3270.6 G	130(1) ns
3277.6 G	137(1) ns	3283.6 G	138(1) ns	3289.6 G	137(1) ns
3296.2 G	138(1) ns	3302.7 G	136(1) ns	3309.2 G	137(1) ns
3315.7 G	138(1) ns	3322.3 G	137(1) ns	3328.8 G	136(1) ns
3334.8 G	137(1) ns	3341.8 G	137(1) ns	3348.4 G	139(1) ns
3354.4 G	138(1) ns	3360.9 G	135(1) ns	3367.4 G	132(1) ns
3373.9 G	128(1) ns	3380.5 G	114(1) ns		

SI4 Relaxation measurements at low temperature region

4. Spin-lattice relaxation (T_1) at different temperatures

T_1 is measured using inverse-recovery method. The pulse sequence is π-T-π/2-τ-π-τ-echo with fixed τ and varied T. We use π/2 = 8 ns and π = 16 ns pulse for the measurement. τ = 150 ns is chosen for detection. 16-step phase cycling was used to eliminate the contribution of unwanted free-induction decay (FID) and echo signals. The experiment data are shown in **Table S4-1.**

Table S4-1. T_1 experiment data measured at different temperatures

Tempreature	T_1 value	Tempreature	T_1 value	Tempreature	T_1 value
5 K	96(11) ms	8 K	84(11) ms	10 K	21(1) ms
15 K	13(3) ms	20 K	0.9(1) ms	25 K	0.27(3) ms
40 K	30(2) μs	60 K	10(1) μs	80 K	6.9(3) μs

5. Phase memory time (T_M) at different temperature

T_M is measured using Hahn-echo decay method. The pulse sequence is π/2-τ-π-τ-echo with τ varies. We use π/2 = 8 ns and π = 16 ns pulse for the measurement. τ = 150 ns is chosen for initial interval. 16-step phase cycling was used to eliminate the contribution of unwanted free-induction decay (FID) and echo signals. The experiment data are shown in **Table S4-2.**

Table S4-2. T_M experiment data measured at different temperatures

Tempreature	T_M value	Tempreature	T_M value	Tempreature	T_M value
5 K	13.6(4) μs	8 K	16.4(7) μs	10 K	17.2(7) μs
15 K	14.3(7) μs	20 K	10.6(5) μs	25 K	8.4(2) μs
40 K	6.4(2) μs	60 K	1.6(1) μs	80 K	1.2(1) μs

6. Phase memory time (T_M) using dynamic decoupling

T_M is measured using Hahn-echo decay method. The pulse sequence is $\pi/2$-τ-$(\pi$-$2\tau)_{n-1}$-π-τ-echo with τ variation. We use π/2 = 8 ns and π = 16 ns pulse for the measurement. τ = 150 ns is chosen for initial interval. Phase cycling was used to eliminate the contribution of unwanted free-induction decay (FID) and echo signals. The experiment data are shown in **Table S4-3.**

Table S4-3 T_M experiment data measured at different temperatures using dynamic decoupling

CPMG-n	T_M value	CPMG-n	T_M value	CPMG-n	T_M value
CPMG-1	13.6(4) μs	CPMG-2	22.1(1) μs	CPMG-4	29.2(1) μs
CPMG-8	38.5(1) μs	CPMG-16	61(1) μs	CPMG-32	68.0(4) μs

SI5 ESEEM measurements at low temperature region

3-pulse ESEEM is measured using the sequence: π/2-τ-π/2-T-π/2-τ-echo, which measures the intensity of the echo as function of delay time T. We use π/2 = 8 ns and π = 16 ns pulse for the measurement. τ = 146 ns and initial T = 400 ns are chosen for interval. The 3-pulse ESEEM effect strongly depends on the magnetic field, so the 3-pulse ESEEM vs B_0 2D experiment was performed. The deepest field position is at the field of 3290 G as shown below in **Figure S5-1**. The 3-pulse ESEEM measurement with good signal to noise is shown in **Figure S5-2**.

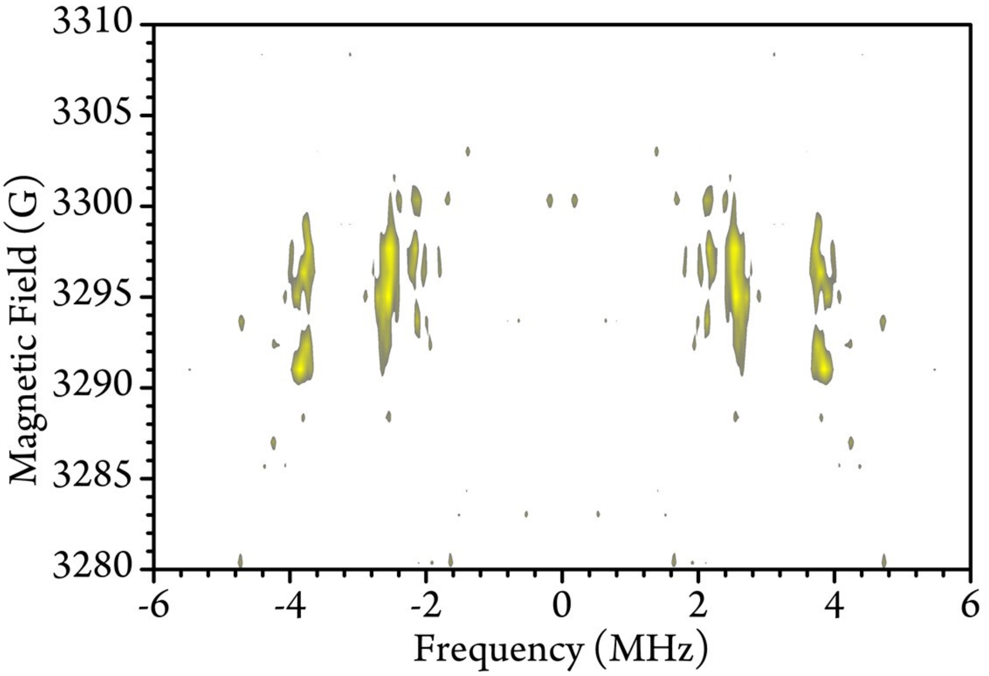

Figure S5-1. The 3pESEEM measurements are performed at different fields. B_0 = 3296 G was selected to excite both allowed and forbidden transitions, therefore it is able to generate the deepest modulation.

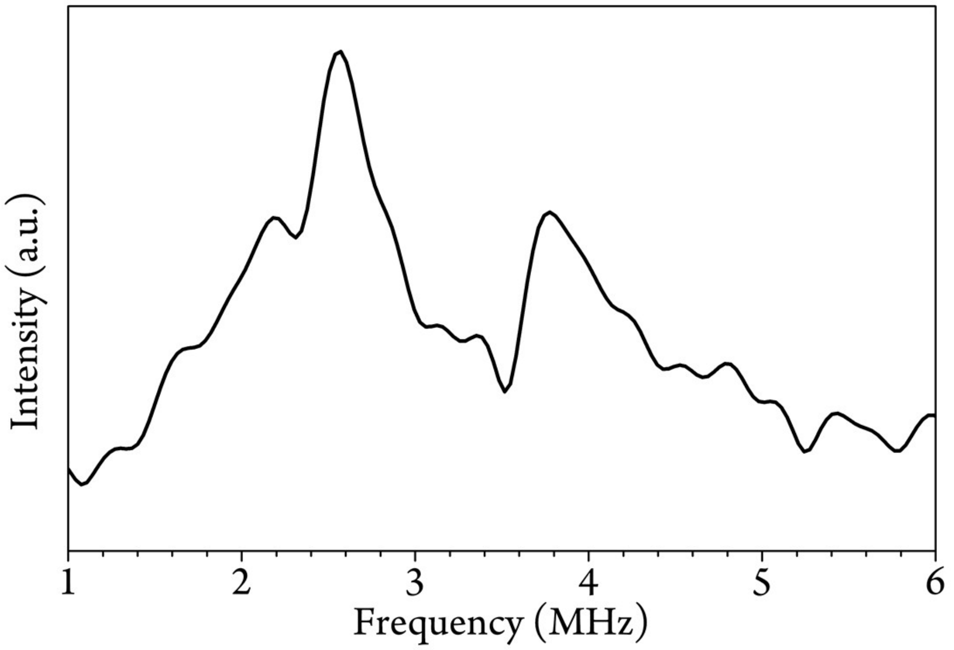

Figure S5-2. The 3pESEEM measurements performed at 3296 G.
